# Prevalence and Clinical Profile of Septic Acute Kidney Injury in Steroid Responsive Nephrotic Syndrome in a Tertiary Care Hospital in Eastern India: A Prospective Observational Study

**DOI:** 10.7759/cureus.89157

**Published:** 2025-07-31

**Authors:** Debanjan Sinha, Piyali Das, Saptarshi Ghosh, Sanat K Ghosh, Mausumi Nandy

**Affiliations:** 1 Pediatrics, Dr. B C Roy Post Graduate Institute of Pediatric Sciences, Kolkata, IND; 2 Obstetrics and Gynaecology, Calcutta National Medical College & Hospital, Kolkata, IND; 3 College of Medicine, Medical College and Hospital, Kolkata, Kolkata, IND; 4 Pediatric Medicine, Dr. B C Roy Post Graduate Institute Pediatric Sciences, Kolkata, IND; 5 Paediatrics, Medical College and Hospital, Kolkata, Kolkata, IND

**Keywords:** acute kidney injury (aki), intensive care unit, nephrotic syndrome, sepsis, septic acute kidney injury (saki)

## Abstract

Background: Acute kidney injury (AKI) and sepsis are well-recognized complications of steroid-responsive nephrotic syndrome. Systemic inflammatory response syndrome (SIRS) initiates inflammation and oxidative stress, which eventually results in septic acute kidney injury (SAKI). A few papers are available in the literature regarding the clinical profile of AKI in nephrotic syndrome; however, there are hardly any data on SAKI in steroid-responsive nephrotic syndrome. Hence, we tried to observe the prevalence and clinical spectrum of SAKI in nephrotic syndrome.

Method:* *A prospective observational study was conducted at DR. B C Roy Post Graduate Institute of Pediatric Sciences, a tertiary care hospital in Kolkata, among children under 12 years of age with steroid-responsive nephrotic syndrome. Patients with any congenital malformation of the kidney and previously associated chronic kidney disease were excluded. EpiInfo 7.2.0.1 of the Centers for Disease Control USA was used for statistical analysis.

Result:* *The total number of participants was 235 nephrotic children. Among 235 participants, 64 (27.23%) nephrotic children developed AKI. Fifty-nine participants out of these 64 children with AKI (92.10%) fulfilled the criteria of SAKI. In the categorization of 59 SAKI patients, 40 (67.8%) children had sepsis, and 19 (32.20%) children had septic shock. Meanwhile, 20.3% (n = 38), 13.6% (n = 12), and 15.3% (n = 9) nephrotic children with SAKI patients developed Stage 1, Stage 2, and Stage 3 AKI, respectively, on Kidney Disease: Improving Global Outcomes (KDIGO) staging. Kidney injury was observed to be more severe with increased severity of sepsis (p-value < 0.000000731). Mortality in SAKI was 8.47%. Duration of hospitalization, requirement of ICU treatment, ventilatory support, and inotrope and renal replacement therapy were more frequent among nephrotic patients developing SAKI.

Conclusion:* * SAKI was found to be the most important complication of steroid-responsive nephrotic syndrome, with higher morbidity and mortality.

## Introduction

Nephrotic syndrome patients are susceptible to infection and sepsis [1-3]. Sepsis induces systemic inflammation, which triggers protective mechanisms within the nephron [4]. Inflammation and oxidative stress in systemic inflammatory response syndrome (SIRS) background activate the host immune system, which results in the production of damage-associated molecular protein (DAMP), pathogen-associated molecular protein (PAMP), reactive nitrogen species (RNS), reactive oxygen species (ROS), and other inflammatory cytokines [5]. In this toxic milieu, the adaptive response of the tubular epithelial cells in the presence of sepsis-associated microvascular dysfunction leads to the development of septic acute kidney injury (SAKI). Although the complex pathophysiology is not completely understood over the decades, in simple terms, SAKI is hypothesized as acute kidney injury (AKI) with no contributory factor for kidney damage other than sepsis [6-7]. However, it is observed that, contrary to renal hypoperfusion in AKI, renal blood flow is found to be normal or increased in SAKI.

Recently, the 28th Acute Disease Quality Initiative (ADQI) work group proposed a new definition of sepsis-associated AKI (SA-AKI) and sepsis-induced AKI (SI-AKI) [8]. They proposed SA-AKI as features of sepsis as per the SEPSIS-3 criteria, along with the presence of AKI as per the Kidney Disease: Improving Global Outcomes (KDIGO) criteria, while AKI features were observed within seven days of sepsis diagnosis. Moreover, SI-AKI was mentioned as a sub-phenotype of SA-AKI, while kidney tissue injury was directly due to sepsis mechanism (excludes nephrotoxic drug usage) [8,9].

Application of the KDIGO shows that one in five adults and one in three children in the hospital setting develop AKI [4]. The morbidity and mortality are also significant in AKI among nephrotic patients [10,11]. Published literature is available about AKI in nephrotic patients, but there is hardly any article related to SAKI in nephrotic patients. A considerable fraction of AKI would be SAKI among nephrotic children. With such hypotheses, the present study was conducted. The objective of this study was to evaluate the prevalence, clinical profile, morbidity, and mortality of SAKI in pediatric patients with steroid-responsive nephrotic syndrome in the hospital setting.

Our study was presented as a poster in the official conference of the American Academy of Pediatrics in February 2022 and was published as a meeting abstract in Pediatrics [12].

## Materials and methods

This prospective observational study was performed at DR. B C Roy Post Graduate Institute of Pediatric Sciences, a tertiary care hospital in Kolkata. All steroid-responsive nephrotic syndrome patients in the age group of one month to 12 years (as per the institution's norm) were admitted to the hospital from the emergency and outpatient departments from September 2018 to December 2019. Any patient with chronic kidney disease or structural malformation of the urinary tract, either congenital or acquired, was excluded. The participants were followed up for the next six months. The Institutional Ethical Committee of Dr. B C Roy Post Graduate Institute of Pediatric Sciences, Kolkata, issued approval (BCH/ME/PR/: 2960).

Sepsis was diagnosed with the SEPSIS-3 criteria [13], and AKI was diagnosed with the KDIGO criteria [14,15]. Duration of intensive care unit (ICU) stay, inotrope requirement, ventilatory support, and renal replacement therapy were taken as morbidity indicators.

Non-infectious causes of SIRS, namely, burn, trauma, surgery, acute pancreatitis, transfusion reaction, autoimmune diseases, dehydration, and electrical injury, were the possible confounders. Participants with potential confounding factors were excluded on clinical grounds.

Data related to variables of interest were collected by standardized patient assessment and medical records. Following admission of nephrotic syndrome patients, we initially started a daily dose of steroid in the form of oral prednisolone at a 2 mg/kg dose. Those who were responsive to our daily dose of steroid were included for detailed data collection, since our inclusion criteria were steroid-responsive nephrotic syndrome patients, as mentioned. Next, detailed history-taking and clinical examination were performed to be kept as the medical record for analysis. The SOFA score was calculated among all included nephrotic patients to categorize them in Sepsis and Septic Shock based on the SEPSIS-3 criteria [13]. Serum creatinine level was monitored on admission and thereafter, and urine output was measured on a daily basis to diagnose AKI among the included participants. AKI grading was done based on the KDIGO criteria [14,15]. SAKI was diagnosed by the simultaneous presence of both sepsis and AKI.

Data on other lab parameters were collected, such as complete blood count, liver function test, kidney function test, urine routine and microscopy, inflammatory markers such as CRP, markers for coagulation disorder like PT, aPTT, INR, ascitic fluid analysis, blood culture, urine culture, ascitic fluid culture details, chest X-ray, USG whole abdomen, and USG-Doppler in suspicion of deep vein thrombosis.

The incidence of SAKI is not available in the literature. However, the incidence of AKI (including septic and non-septic) in nephrotic syndrome, available in various studies, is 20-30% [10,11,16-18]. Assuming an incidence of 20%, the required sample size could be 246 for AKI in nephrotic syndrome. However, our study was on SAKI, which is one kind of AKI. Hence, the sample size of SAKI in nephrotic syndrome was expected to be lower than the sample size of AKI in nephrotic syndrome. In the present study, the sample size was taken as 235. 

In this prospective observational study, information bias was minimized by defining variables with appropriate criteria as mentioned above. Sampling bias was removed by applying inclusion and exclusion criteria. Confounding bias was diminished by excluding subjects having confounding factors.

The incidence and severity of SAKI were expressed in percent. Requirements of intensive care, inotrope therapy, ventilator support, and renal replacement therapy were also expressed in percent, and their durations were expressed by mean with standard deviation where they were applicable.

Regarding the management of sepsis, we have started intravenous ceftriaxone at a 100 mg/kg/day dose to those nephrotic patients who were symptomatic on admission. We have selected this antibiotic and its dose depending on the antibiogram profile of our department. Later, we changed this regimen following laboratory reports and culture-sensitivity results of urine, blood, and ascitic fluid. We predominantly used IV amoxiclav 60 mg/kg/day, IV meropenem 60 mg/kg/day, and IV vancomycin 40 mg/kg/day, depending on the clinical scenario.

Epi Info 7.2.0.1 of the Centers for Disease Control, USA, was used in the statistical analysis of the collected data. Numerical variables were summarized as mean with standard deviation for a normal distribution. Counts and percentages were summarized for categorical variables. Normally distributed numerical variables were compared with Student’s independent sample t-test. Chi-square test or Fisher’s exact test, as appropriate, was used for unpaired proportions for categorical variables. The null hypothesis was rejected in favor of the alternative hypothesis if the calculated p-value was below the threshold chosen. A p-value ≤ 0.05 was considered statistically significant.

## Results

There were a total of 238 children with nephrotic syndrome with a history of steroid responsiveness in the past. However, three patients were excluded due to confounding factors, like two patients with dehydration and one patient as a post-surgical case. Hence, a total of 235 steroid-responsive nephrotic children participated in this study, meeting the inclusion and exclusion criteria.

**Figure 1:**
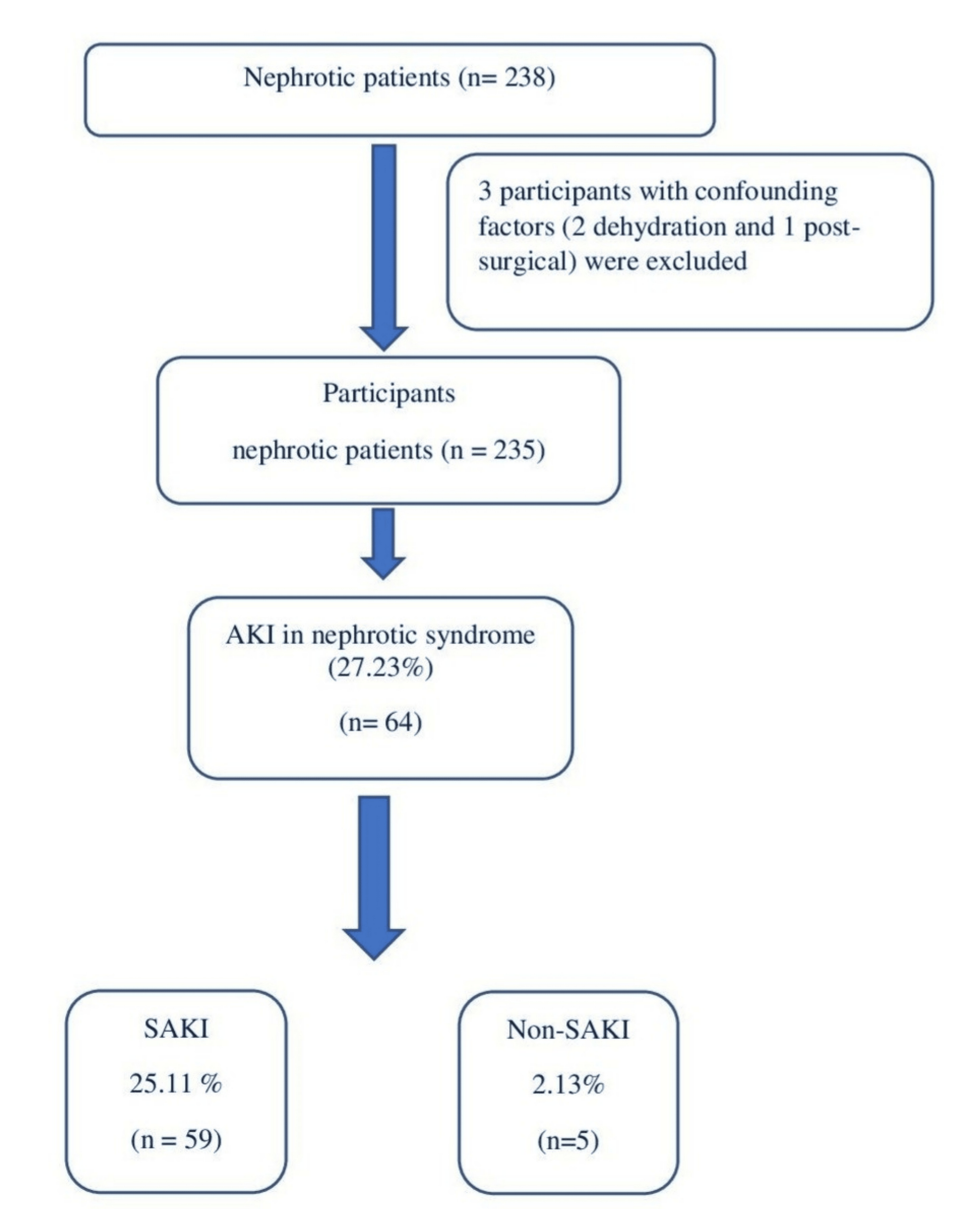
Flow diagram of all the participants in the study AKI: acute kidney injury, SAKI: septic acute kidney injury All data expressed in both numbers and percentage.

Sixty-four patients (27.23%) developed AKI. Out of these 64 AKI patients, 59 had SAKI, and five patients had non-septic AKI. The proportion of SAKI out of all AKI was 92.19%. The incidence of SAKI in steroid-responsive nephrotic syndrome was 25.11%, while the incidence of non-septic AKI (non-SAKI) was 2.13% (Figure 1). Among 59 SAKI patients, five (8.47%) patients expired, with a survival rate of 91.53%.

Requirement of ICU care, ventilatory support, inotrope support, and renal replacement therapy were taken as the morbidity measures of SAKI, as shown in Table 1. Fifty-four participants required ICU care (91.52%) for a mean duration of 12. 86 ± 3.2381 days, while 12 participants needed ventilatory support (20.34%) for a mean duration of 10.8321 ± 3.2378 days. Inotrope support was required in 50 patients (84.75%) for a mean duration of 10.2365 ± 3.2378 days, and seven patients (11.86%) required dialysis for a mean duration of 75.4286 ± 21.5936 hours.

**Table 1:** Requirement of supportive care among steroid responsive nephrotic patients developing septic acute kidney injury (SAKI) The data in the second column has been presented as number and percentage (%). The data in the third column has been presented  as mean ± SD. The data in the fourth and fifth columns has been presented as number and percentage (%).

Supportive care for SAKI	Number (percentage) of participants	Duration (mean value± SD)	Discharged (number (percentage))	Death (number (percentage))
ICU stay	54 (91.52%)	12.86 ±3.2381 [days]	49 (90.74%)	5 (9.26%)
Ventilatory support	12 (20.34%)	10.8321 ± 3.2378 [days]	7 (58.33%)	5 (41. 67%)
Inotrope use	50 (84.75%)	10.2365 ± 3.2378 [days]	45 (90.00%)	5 (10.00%)
Peritoneal dialysis	7 (11.86%)	75.4286 ± 21.5936 [hours]	2 (28.57%)	5 (71.43%)

Among the SAKI patients, 50 participants underwent central venous catheterization for inotrope administration. However, due to proper asepsis precautions, there was no event of central line-associated bloodstream infection (CLABSI). 

Among the 235 participants, five (2.13%) expired. All the mortality was limited to SAKI. Requirement of intensive care, inotrope use, ventilatory support, and need for dialysis were significantly higher in the SAKI group compared to nephrotic syndrome patients who did not develop SAKI (Table 2).

**Table 2:** Morbidity in SAKI and non-SAKI subjects of steroid responsive nephrotic syndrome SAKI: septic acute kidney injury This table shows a comparison of morbidity factors in the SAKI and non-SAKI groups. Comparison of morbidity factors is tabulated, and a Chi-square test is performed to calculate the p-value. Chi-square value added in the fifth column. A p-value < 0.05 was considered significant. Column 1 represents SAKI participants with morbidity factors expressed in number and percentage; duration of each factor expressed as mean value ± SD. Column 2 represents the non-SAKI participants with morbidity factors expressed in number and percentage; duration of each factor expressed as mean value ± SD.

Morbidity profile	Nephrotic children developed SAKI (n = 59)	Nephrotic children, not developing SAKI (n= 176)	Chi-square value χ^2^	p-value
Requirement of intensive care (n)	54 (91.53%)	12 (06.82%)	134.58	<0.0000001
Mean duration (days) of stay in the intensive care unit	12.8631 ± 3.2381	5.8169±1.8249		
Requirement of inotropes (n)	50 (84.75%)	4 (2.27%)	136.81	<0.00001
Mean duration (days) of inotrope use	10.2365 ± 3.2378	4.5 ± 0.5		
Requirement of ventilator (n)	12 (20.34%)	2 (1.14%)	20.55	<0.00001
Mean duration (days) of ventilator support	10.8321 ± 3.2378	4.5 ± 0.5		
Requirement of peritoneal dialysis(n)	7 (1.19%)	1 (0.57%)	11.49	<0.00001
Mean duration (hours) of dialysis	75.4286 ± 21.5936	48		

As per the SEPSIS-3 criteria, 50 patients were grouped in sepsis (84.75%) and nine patients were in septic shock (15.25%). Among the 59 SAKI patients, 38 were in AKI stage 1, 12 in AKI stage 2, and nine in AKI stage 3. Table 3 describes the distribution of sepsis and AKI. It clearly shows that kidney injury is more severe with the progression of sepsis to septic shock.

**Table 3:** Relation of SEPSIS-3 category and severity of AKI AKI: acute kidney injury Participants with sepsis and septic shock are expressed in number along with percentages in the two rows. They are categorized in AKI-I, AKI-II, and AKI-III, and values are expressed in numbers along with percentages. Chi-square statistic (χ²) value: 23.10; degrees of freedom (df): 2; p-value: 0.0000096.

SEPSIS-3 category	Stage 1 AKI ( n= 38)	Stage 2 AKI (n = 12)	Stage 3 AKI (n = 9)	Total
Sepsis (n = 59)	37 (97.37%)	10 (83.33%)	3 (33.33%)	50 (84.75%)
Septic shock (n = 59)	1 (2.63%)	2 (16.67%)	6 (66.67%)	9 (15.25%)
Total (n = 59)	38 (64.41%)	12 (20.34%)	9 (15.25%)	59

Clinical features

**Figure 2:**
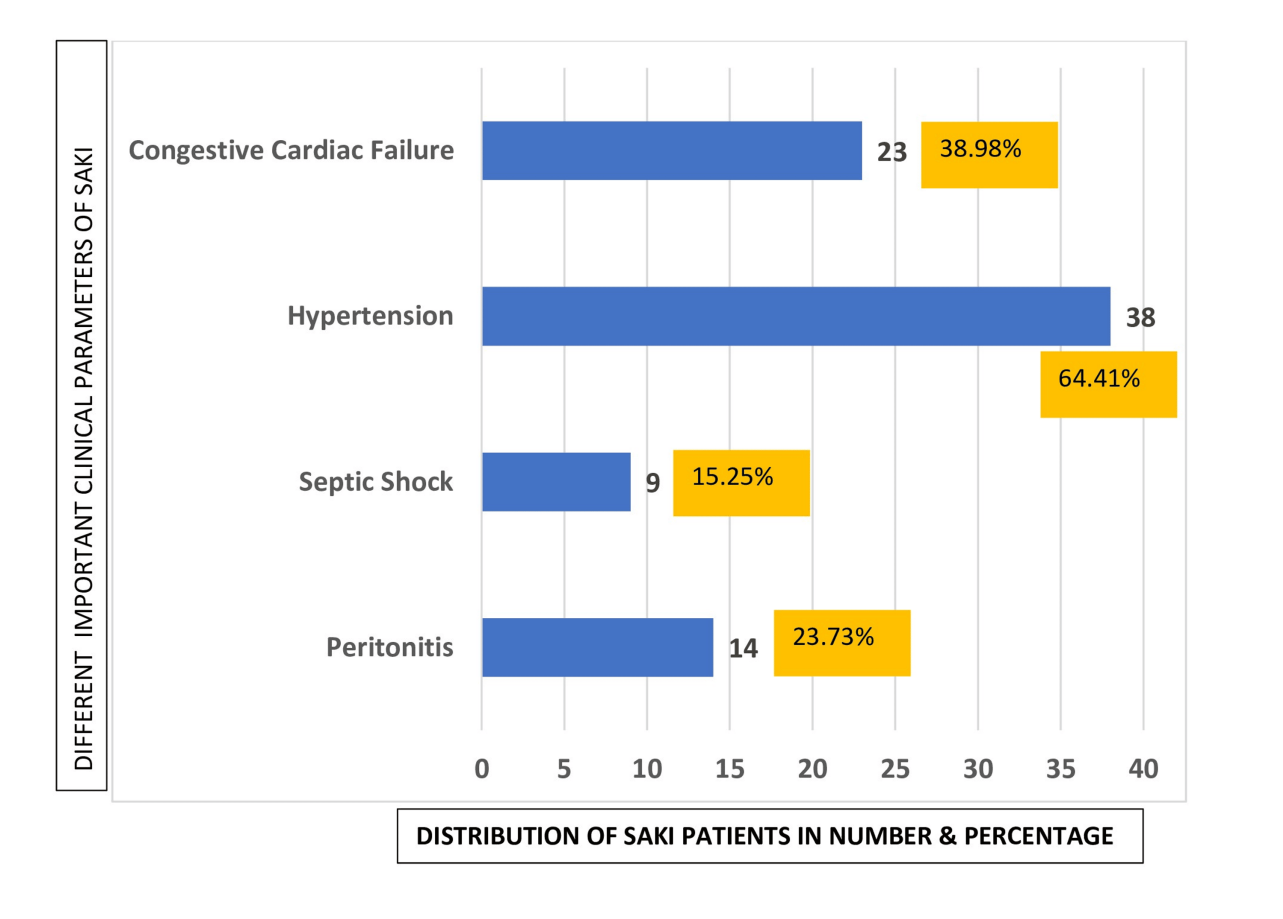
Clinical parameters of SAKI in steroid-responsive nephrotic syndrome SAKI: septic acute kidney injury X-axis: distribution of SAKI patients (both number and percentage shown); Y-axis: different important clinical parameters of SAKI (name of parameters mentioned separately)

Fifty patients of SAKI (84.75%) who did not develop septic shock had prolongation of the oliguric phase, likely due to superimposition of the oliguric phase of nephrotic syndrome and the oliguric phase of AKI. Hypertension was found during illness in 38 patients (64.41%). Congestive cardiac failure was present in the oliguric phase in 23 patients (38.98%) (Figure 2).

Septic shock developed in the remaining nine (15.25%) SAKI patients. After correction of circulatory insufficiency with treatment, these patients developed prolongation of the oliguric phase and hypertension with features of AKI. Meanwhile, 23.73% of SAKI patients (n = 14) developed features of peritonitis.

Predominant complications observed among 235 participants, as shown in Table 4, were peritonitis, sepsis, sepsis with SAKI, cellulitis, and deep vein thrombosis. Five patients expired. All the mortality occurred in the nephrotic syndrome participants who developed SAKI. Morbidity is also increased significantly in this group. Hence, it appears that the sepsis-SAKI sequence is likely to be one of the most dreadful obstacles of steroid-responsive nephrotic syndrome.

**Table 4:** Complications developed among nephrotic syndrome patients in the present study AKI: acute kidney injury Participants with complications are expressed with morbidity and mortality factors in numbers and percentages.

Complications	Frequency	Percentage (N = 235)	ICU requirement	Ventilator requirement	Inotrope requirement	Discharged	Death
Peritonitis	14	5.96 %	10 (71.43%)	0	0	14 (100%)	0
Sepsis without AKI	12	5.11 %	1 (8.33%)	1 (8.33%)	1 (8.33%)	11 (91.67%)	1 (8.33%)
Sepsis + AKI	59	25.11 %	59 (100%)	50 (84.75%)	59 (100%)	54 (91.53%)	5 (8.47%)
Cellulitis	2	0.85 %	0	0	0	2 (100%)	0
Deep vein thrombosis	2	0.85 %	2	0	0	2 (100%)	0

**Figure 3:**
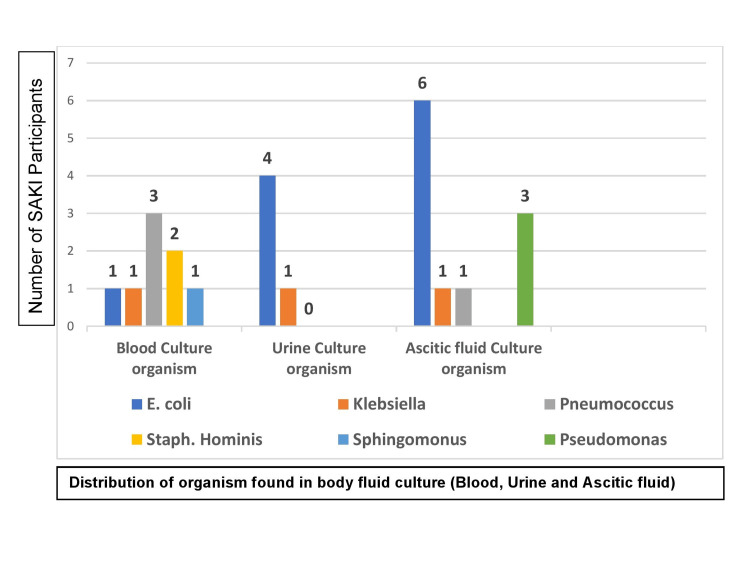
Blood, urine, and ascites fluid organisms in SAKI SAKI: septic acute kidney injury X-axis: distribution of organism found in body fluid culture (blood, urine, and ascites fluid); Y-axis: number of SAKI participants

Urine and blood cultures were done among the SAKI cases to evaluate the causative organism of sepsis (Figure 3). Ascitic fluid was also cultured in cases of peritonitis developed among SAKI patients. Pneumococcus was found as the most prevalent organism (three out of eight SAKI patients) in blood culture (37.5%), while *E. coli *was the most common in both urine (four out of five, i.e., 80%) and ascites fluid (six out of 11, i.e., 54.55%).

## Discussion

In the present study, the incidence of AKI in steroid-responsive nephrotic syndrome was observed to be 27.23% (n = 64), while the incidence of SAKI in nephrotic syndrome was 25.11% (n = 59). The proportion of SAKI patients among all AKI patients with nephrotic syndrome was 92.19% (59 out of 64 AKI participants). Among SAKI patients, 84.75% (n = 50) were in the sepsis group and 15.25% (n = 9) were in the septic shock group. Moreover, 64.41% (n = 38), 20.34% (n = 12), and 15.25% (n = 9) of AKI belonged to KDIGO stage 1, stage 2, and stage 3, respectively. The severity of AKI was higher with the severity of sepsis. Morbidity and mortality were more when nephrotic patients developed SAKI.

The reported overall prevalence of AKI is found to be in the range of 18-32% [16-18] among hospitalized nephrotic patients. However, categorization of AKI into SAKI and non-SAKI was not done in these studies. As a result, the true prevalence of SAKI and the ratio between SAKI and non-SAKI in nephrotic syndrome could not be found from these studies.

It is reported that infection is a common trigger for the development of AKI in nephrotic patients [17]. It is also reported that sepsis, septic shock, spontaneous bacterial peritonitis, and acute gastroenteritis are etiological factors for the development of AKI [18]. Sepsis is not documented in these studies to define SAKI as it is done in our study.

Prasad BS et al. [19] showed that 38%, 16% and 46% of AKI in nephrotic patients developed KDIGO stage 1, stage 2, and stage 3 disease, respectively. In another study, Kushwah et al. [20] showed that 12%, 24%, and 64% of AKI in nephrotic subjects developed stage 1, stage 2, and stage 3 disease on the KDIGO classification. In both studies, AKI was not categorized into SAKI and non-SAKI. The severity of infection was also not categorized into sepsis and septic shock to determine the relation between the severity of sepsis and KDIGO stage AKI. In our study, the severity of sepsis is categorized into sepsis and septic shock by SEPSIS-3. The relation between the severity of infection and KDIGO stage of AKI could be determined.

Morbidity and mortality are much higher in reported studies [19,20] when nephrotic patients develop AKI. This observation is also found in our study, but which subgroup of AKI (SAKI or non-SAKI) that is contributing to increased morbidity and mortality could not be determined from these studies, as AKI is not classified into SAKI and non-SAKI. In our study, it was found that the majority of AKI in nephrotic subjects are SAKI in nature, and increased morbidity and mortality are mostly contributed by the SAKI group.

The limitations of the present study are as follows: i) It was a single-center study with a small sample size. ii) Routine pneumococcal vaccine was not practiced in this study population. Thus, the study population represents the population of developing countries where the pneumococcal vaccine is not routinely practiced. iii) In the present study, we have discussed SAKI with respect to prevalence and clinical profile. However, the management of SAKI is also challenging compared to AKI without sepsis. The outcome of SAKI patients depends on proper control of sepsis. We could not discuss the treatment protocol strategies in the current study. iv) A number of risk factors for AKI in nephrotic syndrome have been reported in the current literature. Sepsis is one of them. Pathophysiology and the clinical spectrum are different in SAKI compared to non-septic AKI. A single prospective study regarding SAKI cannot validate this data due to the lack of a proper comparator group. Comparative future study between SAKI and non-septic AKI in nephrotic syndrome to discuss their clinical features, complications, morbidity, and mortality, and most importantly, the management strategies might establish a strong correlation between SAKI and nephrotic syndrome. This eventually may help to reduce the overall morbidity and mortality of acute kidney injury in nephrotic syndrome. v) Follow-up was not focused on that much in the present study since this was a time-bound study. Hence, a longer follow-up is needed to comment on the long-term complications of SAKI in steroid-responsive nephrotic syndrome. vi) Confounding factors were eliminated as much as possible. However, it is very difficult to rule out all the confounding factors because some factors are responsible for developing SAKI and also non-septic AKI.

In spite of the limitations mentioned above, our study will act as a foundation for future studies on this relatively unexplored aspect of steroid-responsive nephrotic syndrome. Further multicentric studies with an adequate sample size may be conducted with the correction of all limitations of the present study. 

## Conclusions

The prevalence of SAKI is considerable (25.11%) among steroid-responsive nephrotic syndrome patients. The majority of AKI (92.19% of AKI) found in nephrotic syndrome patients were SAKI in nature. Clinical features included the development of sepsis with or without preceding peritonitis, followed by the appearance of features of AKI in an active nephrotic state. The development of SAKI in nephrotic patients increases morbidity and mortality considerably. 
